# Diagnostic Pitfalls of Ruptured Ovarian Serous Borderline Tumors by Minor Trauma: A Case Report

**DOI:** 10.7759/cureus.91058

**Published:** 2025-08-26

**Authors:** Motohiro Yamada, Shoji Oura, Arito Kaji

**Affiliations:** 1 Department of Emergency Medicine, Kishiwada Tokushukai Hospital, Kishiwada, JPN; 2 Department of Surgery, Kishiwada Tokushukai Hospital, Kishiwada, JPN

**Keywords:** massive ascites, minor trauma, ovarian serous borderline tumor, tumor rupture, weight gain

## Abstract

A 54-year-old woman visited our hospital due to abdominal pain after a fall. Computed tomography (CT) showed abdominal distention, massive ascites, and membranous structures of unknown origin, but no mass lesions in the abdomen. Blood tests showed increases in CRP to 31 mg/dL, CA19-9 to 2100 U/mL, and CA125 to 10020 U/mL. Ascitic fluid analysis showed no malignant cells, an increased cell count, and an elevated adenosine deaminase level of 174 U/L. Positron emission tomography showed no avid accumulation of fluorodeoxyglucose in the abdomen. We, therefore, drained the ascites for symptom relief, which unintentionally resulted in marked decreases in CA19-9 and CA125 to 221 U/mL and 1802 U/mL, respectively. Thereafter, an exploratory laparotomy showed a large amount of dirty ascites, abscess, fibrin clots, and no ovarian tumors. A cell block analysis of the ascites taken during the operation showed positivity of CK7, ER, and PAX8 and negativity of CK20 and CDX-2, leading to the diagnosis of a possible uterine or ovarian tumor. The second operation, six weeks after the exploratory laparotomy, showed multiple peritoneal implant-like lesions and a large ovarian tumor with a capsule, which released massive fluid of 6.6 L due to the capsule rupture during the operative procedures. Postoperative pathological study showed that the ovarian tumor was a unilocular cystic tumor with no solid components, had partially thickened cyst walls with hyalinization, and scant presumed invasion of atypical cells in a tubular fashion, finally making the diagnosis of ovarian serous borderline tumor (SBT). The patient recovered uneventfully and has been well without any recurrence for two years. Emergency physicians should note that ovarian SBTs can rupture even with minor trauma and cause underdiagnosis due to the lack of evident solid parts in the tumor.

## Introduction

Ovarian cancer is the second most common gynecologic cancer following the uterine cancer [1]. Although uterine cervical cancer and several luminal cancers, such as gastric and colon cancers, can be pathologically diagnosed through either direct tissue sampling or endoscopic biopsy before operation, ovarian cancer needs a percutaneous needle biopsy for the preoperative pathological diagnosis. Ovarian cancer, therefore, can develop intra-abdominal dissemination with the transcutaneous needle biopsy, often leading to the lack of a pathological diagnosis before ovarian cancer operation.

Ovarian tumors do not have any specific symptoms and can develop various symptoms with tumor oppression or invasion [2]. Some ovarian tumors that generate serous or mucinous fluid can cause abdominal distention and give the patient weight gain. Some patients, however, do not realize the fluid accumulation, if it is slow, and only recognize it as weight gain due to becoming obese.

Among various ovarian tumors, it is often difficult for diagnostic physicians to distinguish between serous borderline tumors (SBTs) [3] and low-grade serous carcinomas (LGSCs) [4], even by pathological evaluation, and naturally much more difficult by image diagnosis. Oncologists, therefore, can generally get a pathological diagnosis of SBTs only after the tumor removal.

SBTs and LGSCs generally show favorable clinical outcomes, have large cystic parts, and can be easily treated with surgery. Diagnosis becomes even more difficult when the cystic portion of these diseases is ruptured by abdominal trauma. In short, symptoms associated with abdominal trauma and atypical images due to cyst contents spillage can make it more difficult for not only diagnostic physicians but also for emergency physicians to diagnose and manage these disorders appropriately.

We herein report an extremely rare minor trauma-induced ruptured SBT, which annoyed us even with the judgment for the presence of an ovarian tumor itself.

## Case presentation

**Figure 1:**
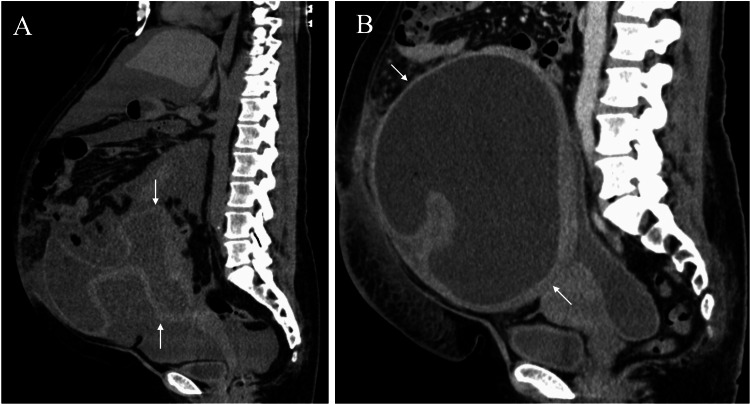
Computed tomography (CT) findings A. Non-contrast sagittal abdominal CT two weeks after the initial consultation showed massive ascites, membranous structures of unknown origin (arrows), and no evident masses. B. Non-contrast sagittal abdominal CT six weeks after the initial consultation showed a large cystic lesion (arrows).

A 54-year-old woman visited our hospital due to abdominal pain after a forward fall. The mildness of the abdominal pain led the emergency physician to only prescribe painkillers and cold compresses without performing any image evaluation. Appetite loss and abdominal distention made her revisit our hospital three days later. She had been aware of gradual abdominal distention for the past 10 years, but had not visited any hospitals because she had judged it to be a symptom of obesity. Computed tomography (CT) showed abdominal distention, massive ascites, and membranous structures of unknown origin but no mass lesions in the abdomen (Figure 1).

**Figure 2:**
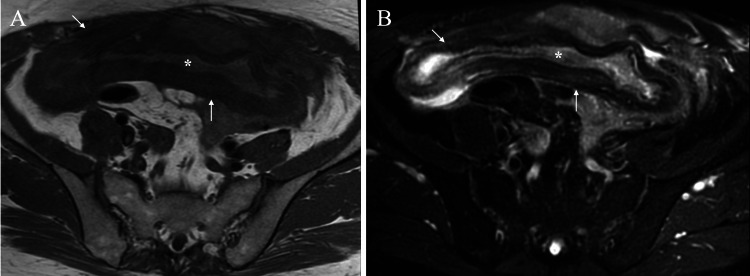
Magnetic resonance imaging (MRI) findings A. T1-weighted images of both the presumed collapsed capsule (arrows) and its internal contents (asterisk) showed low signals. B. Fat-suppressed T2-weighted images showed low signals in the membranous structures (arrows) and high signals in their contents (asterisk).

Blood tests showed normal carcinoembryonic antigen (CEA) and α-fetoprotein levels but increases in C-reactive protein (CRP) to 31 mg/dL, CA19-9 to 2100 U/mL (reference range 0-37 U/mL), and CA125 to 10020 U/mL (reference range 0-35 U/mL). Ascitic fluid analysis showed no malignant cells, an increased cell count, and an elevated adenosine deaminase level of 174 U/L (reference range 8.6-20.5 U/L). Unlike CT findings, magnetic resonance imaging showed that the membranous structures seemed to be a ruptured capsule (Figure 2).

**Figure 3:**
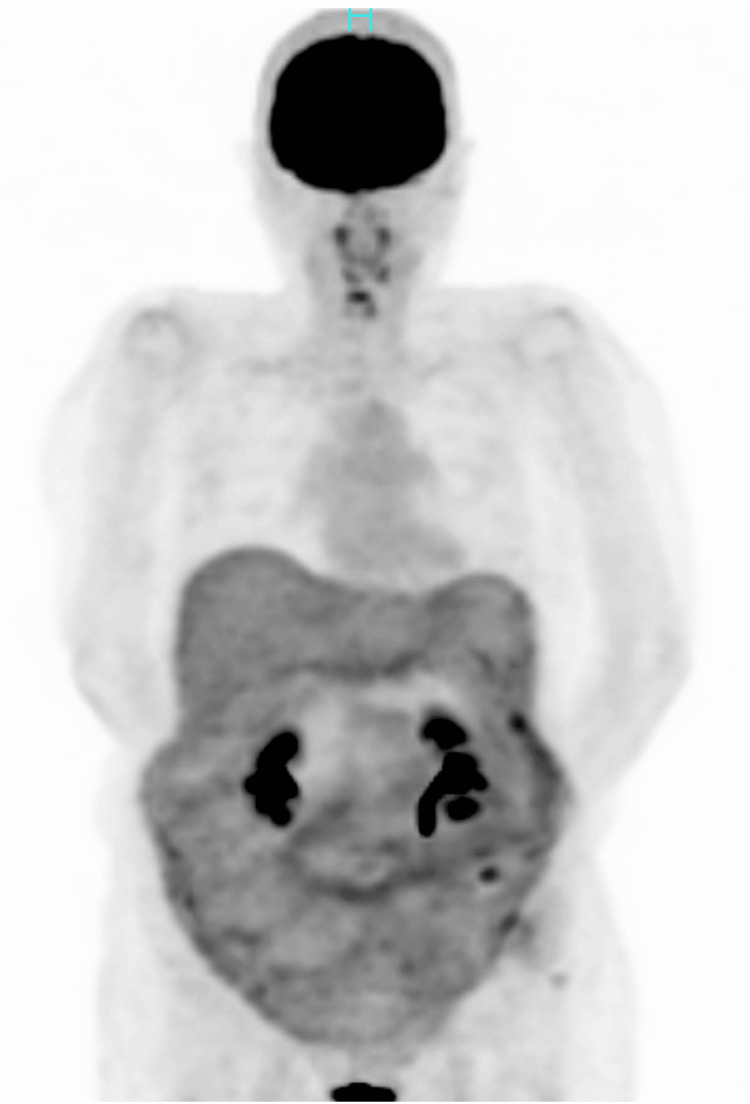
Positron emission tomography (PET) PET showed no avid fluorodeoxyglucose uptake in the abdomen.

Positron emission tomography showed no avid accumulation of fluorodeoxyglucose in the abdomen (Figure 3).

These findings, except for capsule-like structures, made us judge that the ascites was due to secondary bacterial peritonitis and drain the ascites for symptom palliation, leading to symptom relief and unintentional marked decreases of elevated CA19-9 and CA125 levels to 221 U/mL and 1802 U/mL, respectively (Table 1).

**Table 1:** Blood tests AFP: alpha-fetoprotein, ALP: alkaline phosphatase, ALT: alanine aminotransferase, AST: aspartate aminotransferase, CEA: carcinoembryonic antigen, Ch-E: cholinesterase, CK: creatine kinase, CRP: C-reactive protein, γ-GTP: γ-glutamyl transpeptidase, Hb: hemoglobin, Ht: hematocrit, LDH: lactate dehydrogenase, RBC: red blood cell count, WBC: white blood cell count

Test	Reference range	Initial presentation	Two weeks after ascites drainage	Two months after ascites drainage
Total bilirubin	0.4-1.5 mg/dL	1.8	0.7	0.4
Direct bilirubin	0.1-0.3 mg/dL	0.74	-	0.1
AST	13-30 U/L	13	14	22
ALT	7-23 U/L	11	15	28
LDH	124-222 U/L	260	169	234
ALP	38-113 U/L	78	47	93
γ-GTP	9-32 U/L	25	18	26
Ch-E	201-421 U/L	176	119	115
CK	41-153 U/L	49	18	29
Amylase	44-132 U/L	31	28	40
Total protein	6.6-8.1 g/dL	6.1	4.9	7.1
Albumin	4.1-5.1 g/dL	2.7	2.3	2.8
CRP	0-0.14 mg/dL	31	3.7	4.7
WBC	33-86 × 10^2^/μL	103	127	75
RBC	386-492 × 10^4^/μL	422	475	383
Hb	11.6-14.8 g/dL	11.7	13.0	10.2
Ht	35.1-44.4 %	36	40.7	32.5
Platelet	15.8-34.8 × 10^4^/μL	30.1	48.7	43.7
CEA	0-5.0 ng/mL	0.9	0.9	1.1
AFP	0-7.0 ng/mL	1.0	6.4	-
CA19-9	0-37 U/mL	2100	221	13.8
CA125	0-35 U/mL	10020	1802	91.5

**Figure 4:**
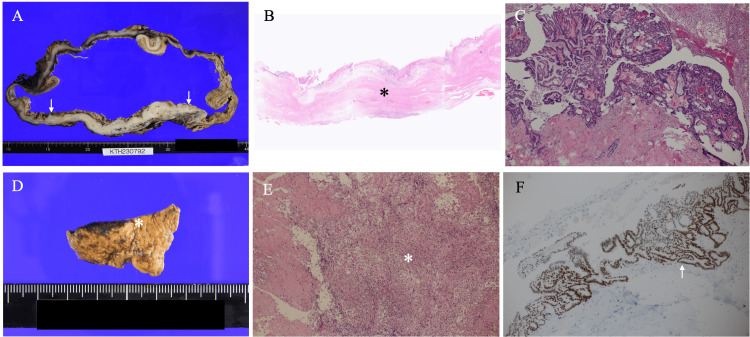
Pathological findings A. Cut surface of the tumor showed that the tumor had a capsule with partially thickened parts (arrows) and no evident solid masses. B. Non-thickened capsule parts had predominantly fibrous components (asterisk). C. The fibrous capsule had cuboidal to columnar tumor cells proliferating in a papillary fashion (asterisk) and small invasive components in a tubular fashion (arrow). D. The peritoneal implant-like lesions were brown to skin-colored and had clear margins. E. The peritoneal implant-like lesions had numerous histiocytes (asterisk). F. Tumor cells were positive for estrogen receptor (arrow).

Cell block analysis of the ascites further showed negativity of calretinin, estrogen receptor (ER), and PAX8, and positivity of CD68 [4-6], highly suggesting no evidence of possible malignancy. Therefore, to investigate the etiology of massive ascites, we performed an exploratory laparotomy on the patient on the 18th day after hospitalization. We, however, unfortunately, could not find ovaries, but found abscess, fibrin clots, and a large amount of dirty ascites, consistent with peritonitis. A repeat cell block examination of the ascites taken during the operation showed positivity of CK7, ER, and PAX8 and negativity of CK20 and CDX-2, leading to the diagnosis of a possible uterine or ovarian tumor. Based on these immunostaining results and image findings, we determined that the tumor was an ovarian tumor and attempted to resect it 6 weeks later. The operation showed a large left ovarian tumor with a capsule, which released massive fluid of 6.6 L due to the rupture of the capsule during the operative procedures. We further identified multiple peritoneal implant-like lesions after the removal of the left ovarian tumor. We, therefore, resected the left ovarian tumor and all peritoneal implant-like lesions without greater omentum resection. Postoperative pathological study showed that the ovarian tumor was a unilocular cystic tumor with no solid components, had necrotic components with bleeding in its large cyst, and granulomatous tissue mainly consisting of fibroblasts, cholesterin crystals, histiocytes, and inflammatory cells. The cyst walls had cuboidal to columnar atypical cells growing in a papillary fashion, hyalinization, and scant presumed invasion of atypical cells in a tubular fashion. Peritoneal implant-like lesions had numerous cytoplasm-rich histiocytes, fibroblasts, cholesterol crystals, and foreign body-type multinucleated giant cells. The former two pathological components had neither nuclear atypia nor mitoses, which made the peritoneal implant-like lesions be judged as xanthogranulomatous inflammation. We, therefore, diagnosed the resected ovarian mass as an ovarian SBT (Federation of Gynecology and Obstetrics (FIGO) stage IA) (Figure 4).

The patient recovered uneventfully and has been well without any recurrence for two years.

## Discussion

Benign or low-grade ovarian tumors generally grow slowly and make patients visit hospitals late, often leading to huge ovarian tumor formation [7,8], like this case. We can easily speculate that the ovarian tumor in this case was extremely large due to the following three findings. First, a CT taken three days after the abdominal trauma showed massive fluid spreading throughout the abdomen and caused abdominal distention. Second, 6.6 L of aspirated ascites was confirmed at the second surgery. And lastly, the patient's weight of 100 kg on admission became 80 kg just after the ovarian tumor resection.

It is well known that prolonged compression by tumors can cause xantogranulomatous inflammation, i.e., cholesterol granuloma [9]. Diagnostic physicians can hardly distinguish between cholesterol granulomas and SBTs/LGSCs by images. In fact, we judged that the peritoneal lesions were peritoneal implants rather than cholesterol granulomas during the operation.

Tumor markers, i.e., CA125 and CA19-9, showed abnormally high values in this case, which could not contribute to determining this disorder to be malignant. Simple drainage of the massive ascites markedly reduced the CA125 levels from 10020 U/mL to 1802 U/mL and the CA19-9 levels from 2100 U/mL to 221 U/mL, respectively. It, therefore, seems reasonable to judge that increases in tumor markers in this case were not by the ovarian cancer, but by the peritoneal tumor and inflammation. Diagnostic physicians generally think CA125 is a tumor marker of ovarian cancer [10]. It, however, is well known that CA125 levels basically reflect mesothelial activity in the pleura and peritoneum. Therefore, large benign peritoneal tumors and widespread peritoneal inflammation can naturally induce CA125 level elevation. CA19-9 is expressed in various organs such as pancreatic ducts, bile ducts, gallbladder, salivary glands, bronchial glands, prostate, stomach, large intestine, and uterine endometrium [11]. In short, large abdominal benign tumors can elevate CA19-9 levels. It, therefore, is reasonable for us to judge that the high tumor marker levels were due to a large peritoneal lesion with inflammation.

We could detect no evident invasive components but a faint presumed invasive part of SBTs in the fibrous capsule, which markedly annoyed pathologists as to whether to judge this tumor to be malignant or not. Ruptured fibrous capsule initially led us to diagnose this disorder not through the differential diagnosis of ovarian tumors but through that of ascites-inducing disorders, leading to no idea of SBT or LGSC as a differential diagnosis despite the confirmation of a presumed fibrous capsule both on CT and MRI. It, therefore, is very important for diagnostic physicians to note that both SBTs and LGSCs generally lack solid components. 

In hindsight, early recognition of the fibrous capsule as a key diagnostic clue could have expedited the diagnosis. Emergency physicians, including diagnostic physicians, should take SBTs and LGSCs into consideration in differential diagnosis when massive ascites without any evident masses is present with fibrous capsule-like structures.

## Conclusions

SBTs generally have fibrous capsules that can be easily ruptured only by minor trauma. In addition, SBTs generally lack solid components in their capsules, often making emergency physicians annoyed, even with the judgment for the presence of the ovarian tumor itself. Emergency physicians, therefore, should note that membranous structures in the massive ascites after minor trauma to the abdomen are an important diagnostic clue for the differential diagnosis of SBTs.
